# 1209. The Evolving Nature of Syndromic Surveillance During the COVID-19 Pandemic in Massachusetts

**DOI:** 10.1093/ofid/ofab466.1401

**Published:** 2021-12-04

**Authors:** Sarah J Willis, Karen Eberhardt, Liisa Randall, Alfred DeMaria, Catherine M Brown, Lawrence C Madoff, Bob Zambarano, Aileen Ochoa, Michael Klompas, Noelle Cocoros

**Affiliations:** 1 Harvard Medical School and Harvard Pilgrim Health Care Institute, Boston, Massachusetts; 2 Commonwealth Informatics, Waltham, Massachusetts; 3 Massachusetts Department of Public Health, Boston, Massachusetts

## Abstract

**Background:**

We developed a syndromic algorithm for COVID-19 like illness (CLI) to provide supplementary surveillance data on COVID-19 activity.

**Methods:**

The CLI algorithm was developed using the Electronic Medical Record Support for Public Health platform (esphealth.org) and data from five clinical practice groups in Massachusetts that collectively care for 25% of the state’s population. Signs and symptoms of CLI were identified using ICD-10 diagnosis codes and measured temperature.

The algorithm originally included three categories: Category 1 required codes for coronavirus infection and lower respiratory tract infections (LRTI); Category 2 required an LRTI-related diagnosis and fever; Category 3 required an upper or lower RTI and fever.

The three categories mirrored statewide laboratory-confirmed case trends during spring and summer 2020 but did not detect the increase in late fall. We hypothesized this was due to the requirements for fever and LRTI. Therefore, we added three new categories defined by milder symptoms without fever: Category 4 requires LRTI-related diagnoses only; Category 5 requires upper or lower RTI or olfactory/taste disorders; and Category 6 requires at least one sign of CLI not identified by another category.

**Results:**

The six-category algorithm detected the initial surge in April 2020, the summer lull, and the second surge in late fall (see figure). Category 1 cases were not identified until mid-March, which coincides with the first laboratory-confirmed cases in Massachusetts. Categories 2 and 3, which required fever, were prominent during the initial surge but declined over time. Category 5, the broadest category, declined during February and March 2020, likely capturing the end of the influenza season, and successfully detected the spring surge and fall resurgence. Weekly number of COVID-19 like illnesses by category, February 2, 2020 through May 8, 2021

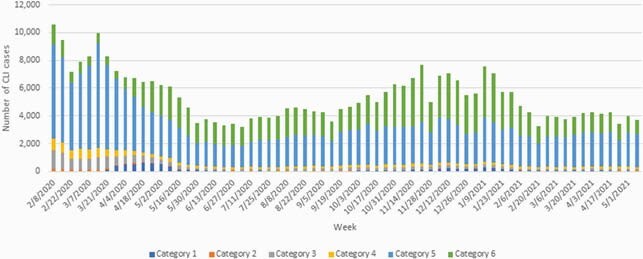

**Conclusion:**

A syndromic definition that included mild upper RTI and olfactory/taste disorders, with or without fever or LRTI, mirrored changes in laboratory-confirmed COVID-19 cases better than definitions that required fever and LRTI. This suggests a shift in medically attended care and/or coding practices during initial vs subsequent surges of COVID-19, and the importance of using a broad definition of CLI for ongoing surveillance.

**Disclosures:**

**Michael Klompas, MD, MPH**, **UpToDate** (Other Financial or Material Support, Chapter Author)

